# Dementia risk reduction assessed with ANU‐ADRI in the *Maintain Your Brain* trial, an online multidomain intervention to prevent cognitive decline and dementia

**DOI:** 10.1002/alz.090586

**Published:** 2025-01-09

**Authors:** Tiffany Chau, Nancy Briggs, Gordana Popovic, Kaarin J. Anstey, Megan Heffernan, Maria A. Fiatarone Singh, Michael Valenzuela, Michael Millard, Perminder S. Sachdev, Nicola T Lautenschlager, Louisa Jorm, Jeewani Anupama Ginige, Henry Brodaty

**Affiliations:** ^1^ Centre for Healthy Brain Ageing (CHeBA), UNSW Sydney, Sydney, NSW Australia; ^2^ UNSW, Sydney, NSW Australia; ^3^ UNSW Ageing Futures Institute, Sydney, NSW Australia; ^4^ George Institute, University of New South Wales, Sydney, NSW Australia; ^5^ University of Sydney, Sydney, NSW Australia; ^6^ University of New South Wales, Sydney, NSW Australia; ^7^ St Vincent’s Hospital, Sydney, NSW Australia; ^8^ Centre for Healthy Brain Ageing (CHeBA), University of New South Wales, UNSW Sydney, NSW Australia; ^9^ The University of Melbourne, Parkville, VIC Australia; ^10^ Western Sydney University, Penrith, NSW Australia

## Abstract

**Background:**

The *Maintain Your Brain* (MYB) randomised controlled trial (RCT) aimed to prevent cognitive decline and dementia through a multidomain risk‐reduction intervention delivered digitally. The intervention targeted four modifiable risk areas (physical inactivity, poor diet, cognitive inactivity, and depression and/or anxiety). MYB ran for three years and targeted older Australians aged 55‐77 years. We previously reported significant benefits on cognition in the intervention group compared to the control group. Here we report the outcome on a validated dementia risk assessment tool.

**Method:**

The self‐reported short form of the Australian National University Alzheimer’s Disease Risk Index (ANU‐ADRI‐SF; 1) was used to assess dementia risk in MYB. To examine risk and protective factors, non‐modifiable factors (age and sex) were removed. Unrealistic values were also removed as part of the data cleaning process. A generalised linear mixed model with appropriate response distributions and link function was run to examine the group (intervention vs control) by time (baseline, 12‐, 24‐, and 36‐months) interaction. An additional analysis adjusted for baseline age, gender, and years of education was run.

**Result:**

Of 14,064 participants who consented, 6,104 participants were eligible and completed baseline assessments. They were randomised 1:1 to the intervention (n = 3,051) or control (n = 3,053) group. Upon removing outliers (n = 58), 6,046 participants were included in the mixed model analysis. Analysis revealed significant intervention effects on dementia risk change over three years (0.56, 95%CI ‐0.88‐ ‐0.30, p<0.001). Results were replicated in the adjusted model (p<0.001).

**Conclusion:**

An online intervention program confirmed results from a 24‐month follow‐up of in‐person multicomponent interventions (2,3) in lowering dementia risk. Overall, MYB has the potential to provide a realistic path towards scalable community‐based prevention of dementia, resulting in an improvement in public health and potential economic implications.

**References**:

1. Kim S et al, *Alzheimers Dement*: *Translational Res & Clin Interventions* 2016;2:93‐98

2. Barbera et al., *Alzheimers Dement J Alzheimers Assoc*. 2020; 16:S10

3. Deckers K et al. *Alzheimers Dement*. 2021;17:1205‐1212.

